# Risk Factor Analysis of Venous Thromboembolism in Cancer: A National Inpatient Sample Study

**DOI:** 10.7759/cureus.24323

**Published:** 2022-04-20

**Authors:** Satya Rijal, Gagan Kaur, Chia Chi Loh, Sowmya Sagireddy, Hadeel Dweik, Moinulhaq P Makrani, Ramya Akella

**Affiliations:** 1 Internal Medicine, Southeast University Medical College, Nanjing, CHN; 2 Medicine and Surgery, Sri Guru Ram Das Institute of Medical Sciences and Research, Amritsar, IND; 3 Internal Medicine, Manipal University College Malaysia, Melaka, MYS; 4 Internal Medicine, Coney Island Hospital, New York, USA; 5 Medicine, The University of Jordan, Amman, JOR; 6 Pharmacology, Parul Institute of Medical Sciences and Research, Waghodia, IND; 7 Internal Medicine, Pikeville Medical Center, Pikeville, USA

**Keywords:** nationwide inpatient sample (nis), medical comorbidities, risk factor for vte, venous thromboembolism (vte), cancer associated vte

## Abstract

Objective

In this study, we aimed to explore the association of demographic characteristics and comorbidities with the risk of venous thromboembolism (VTE) in cancer inpatients, as well as to delineate the mortality risk in cancer inpatients with VTE.

Methods

We conducted a retrospective cohort analysis based on the National Inpatient Sample (NIS) 2012-2014, involving 339,395 inpatients with a primary diagnosis of cancer subdivided into cohorts without VTE (n=331,695) and with VTE (n=7,700). We used a binomial logistic regression model to evaluate the odds ratio (OR) of demographics, comorbidities, and in-hospital mortality rate with respect to cancer inpatients with VTE.

Results

A higher proportion of cancer inpatients with VTE were 36-50 years in age (83.1%), male (50%), and of black (19.3%) and Hispanic ethnicity (17.2%) compared to the non-VTE cohort. The prevalence of comorbidities was higher in the VTE cohort, including HIV/AIDS, congestive heart failure (CHF), chronic pulmonary disease, diabetes, hypertension, and obesity. CHF demonstrated the highest risk of association with VTE (OR: 2.68, 95% CI: 2.30-3.12), followed by hypertension (OR: 1.23, 95% CI: 1.16-1.29), diabetes (OR: 1.16, 95% CI: 1.07-1.26), and chronic pulmonary disease (OR: 1.13, 95% CI: 1.05-1.22). Conversely, valvular diseases, obesity, and drug abuse were not significantly associated with VTE in cancer inpatients. The in-hospital mortality rate was higher in cancer inpatients with VTE (12% vs. 2.1%), thereby increasing the in-hospital mortality risk (OR: 3.87, 95% CI: 3.58-4.18).

Conclusion

VTE risk was significantly higher in cancer patients with comorbid CHF, hypertension, diabetes, and chronic pulmonary disease. The risk of all-cause in-hospital mortality was increased by four times in cancer inpatients with VTE.

## Introduction

Venous thromboembolism (VTE) is a multifactorial disease that involves clot formation in the deep veins of the legs and pelvis, which may lead to fatal complications. VTE affects more than 900,000 individuals in the United States (US), with an annual incidence rate of one to two individuals per 1,000 population [1]. The incidence rate is much higher at eight per 1,000 in individuals who are over the age of 85 years as they are at a two-fold increased risk of developing VTE compared to the young and middle-aged population [1,2].

Cancer is the second leading cause of death in the US, with an estimated 1,806,590 new cases in 2020 and a total of 606,520 cancer-related deaths as per the National Cancer Institute data [3]. Cancer patients are at the highest risk within a year after the initial diagnosis, reducing overall survival rates. Cancer accounts for 20% of the overall incidence of VTE, with one in five blood clots being cancer-related [4]. About 4-20% of cancer patients will experience at least one episode of VTE during their disease course. A five to seven-fold increased risk is seen in patients receiving chemotherapy for over 12 months compared to non-cancer patients, as some chemotherapeutic agents are known to be prothrombotic. Hence, all cancer patients must be periodically assessed for the risk and possibility of VTE [5].

Malignancy associated-VTE is a major complication of cancer, representing the second most frequent cause of death worldwide among cancer patients [6]. The prevalence rate of VTE in cancer patients is higher (about 15%) compared to cancer patients without comorbid VTE [1]. A population-based study showed that cancer patients are at four times higher risk of developing venous thrombosis, and the risk is significantly higher (6.5-fold) among those receiving chemotherapeutic treatment. The prognosis of cancer after thromboembolism has been associated with poor outcomes and adverse impact on the quality of life (QoL) for patients, with an increasing rate of morbidity, hospitalizations, and higher healthcare costs. Also, the mortality risk ratio after one year is 2.2 due to the invasive nature of the tumor and the complications of malignancy-associated thrombosis [7].

The mechanisms of cancer-related thrombosis are unclear, but several hypotheses have been proposed to explain the underlying pathogenesis. Virchow's triad, which comprises hypercoagulability, venous stasis, and endothelial injury, is an important contributor to the development of VTE. Similarly, prothrombotic and hypercoagulability states of malignancy are currently known as driving factors of thrombosis. The direct activation of the coagulation cascade and platelets cause the release of cytokines including tumor necrosis factor-alpha (TNF-a) and interleukin-1b (IL-1b) from the tumor cells, greatly influencing the formation of plasminogen activator inhibitor (PAI-1), which acts as an inhibiting factor for fibrinolysis, indirectly promoting thrombogenesis during malignancy [5].

In light of these facts, it is important to study the prevalence and mortality risk association of VTE in cancer patients as they are predictors of outcomes for complications and may help identify the priorities of healthcare by specifically focusing on treatment and disease prevention in clinical settings. We conducted this study to evaluate the association of demographic characteristics and comorbidities with the risk of VTE in cancer inpatients, as well as to delineate the mortality risk in cancer inpatients with VTE.

## Materials and methods

We conducted a retrospective cohort study based on the largest healthcare-related database in the US, the National Inpatient Sample (NIS), from January 1, 2012, to December 31, 2014. As the NIS includes publicly available de-identified data, we did not require approval from the institutional review board [8]. We included 339,395 adult inpatients (aged 18-50 years) with a primary discharge diagnosis of cancer and subdivided them into two cohorts: patients without VTE (n=331,695) and with VTE (n=7,700). We excluded patients aged above 50 years to reduce the confounding effect of chronic conditions and cardiometabolic comorbidities.

NIS includes several International Classification of Diseases, Ninth Revision (ICD-9) diagnosis codes that are classified into clinically relevant categories by the Clinical Classifications Software (CCS). Hence, cancer was identified using the respective CCS codes as follows: cancer of the head and neck (CCS 11), esophagus (CCS 12), stomach (CCS 13), colon (CCS 14), rectum and anus (CCS 15), liver (CCS 16), pancreas (CCS 17), other gastrointestinal organs (CCS 18), lungs (CCS 19), other respiratory (CCS 20), bone and connective tissue (CCS 21), skin (CCS 22, 23), breast (CCS 24), uterus, (CCS 25), cervix (CCS 26), ovary (CCS 27), other female genital organs (CCS 28), prostate (CCS 29), testis (CCS 30), other male genital organs (CCS 31), bladder (CCS 32), kidney (CCS 33), other urinary organs (CCS 34), brain and nervous system (CCS 35), and thyroid (CCS 36). VTE was identified by the ICD-9 diagnosis codes for lower extremity deep vein thrombosis (DVT) (451.1x, 451.2, 451.81, 453.4x, 453.5x), upper extremity DVT (451.83, 451.84, 451.89, 453.72, 453.73, 453.74, 453.75, 453.76, 453.77, 453.82, 453.83, 453.84, 453.85, 453.86, 453.87), and other venous thromboses (451, 451.9, 452, 453, 453.0, 453.1, 453.2, 453.3, 453.79, 453.8, 453.89, 453.9).

We considered demographic characteristics (age, sex, and race), and comorbidities that were coexisting diagnoses in the inpatient records. The comorbidities included in the study were as follows: HIV/AIDS, congestive heart failure (CHF), valvular diseases, chronic pulmonary diseases, diabetes, hypertension, obesity, and drug abuse. We also included the all-cause in-hospital mortality as an outcome of interest in cancer inpatients as documented in the NIS.

We used descriptive statistics and Pearson’s chi-square test to delineate the differences between cancer inpatients without VTE versus those with VTE. Also, we used the binomial logistic regression model to evaluate the demographic characteristics and comorbidities that increased the odds ratio (OR) of association of VTE in cancer inpatients. We used a binomial logistic regression model to measure the OR of association between VTE and in-hospital mortality and this model was adjusted for age, sex, and race. All analyses were conducted using SPSS Statistics version 27.0 (IBM Corp., Armonk, NY), and statistical significance was set at a two-sided p-value of <0.01.

## Results

Our study sample of 339,395 cancer inpatients predominantly comprised middle-aged adults (36-50 years, 80.3%), females (61.8%), and whites (62.7%). Compared to the non-VTE group, VTE was seen in a significantly higher proportion of middle-aged adults aged 36-50 years (83.1% vs. 80.2%), males (50% vs. 37.9%), blacks (19.3% vs. 13.8%), and Hispanics (17.2% vs. 13.7%). The prevalence of comorbidities was higher in cancer inpatients with VTE compared to the non-VTE group, including HIV/AIDS, CHF, chronic pulmonary disease, diabetes, hypertension, and obesity as shown in Table 1.

**Table 1:** Distribution of cancer inpatients VTE: venous thromboembolism; HIV/AIDS: human immunodeficiency virus/acquired immunodeficiency syndrome

Variable	VTE (no), %	VTE (yes), %	Total, %	P-value
Age				
18-35 years	19.8	16.9	19.7	
36-50 years	80.2	83.1	80.3	<0.001
Sex				
Male	37.9	50.0	38.2	
Female	62.1	50.0	61.8	<0.001
Race				
White	62.9	53.9	62.7	<0.001
Black	13.8	19.3	14.0	
Hispanic	13.7	17.2	13.8	
Other	9.5	9.6	9.5	
Comorbidities				
HIV/AIDS	0.9	1.9	0.9	<0.001
Congestive heart failure	0.8	2.5	0.8	<0.001
Valvular diseases	0.9	1.0	0.9	0.409
Chronic pulmonary diseases	10.1	11.6	10.2	<0.001
Diabetes	7.9	10.8	8.0	<0.001
Hypertension	22.2	29.0	22.4	<0.001
Obesity	11.8	13.1	11.8	<0.001
Drug abuse	2.5	3.0	2.6	0.014

Next, we evaluated the OR of association across various variables in cancer inpatients with VTE by comparing it with the non-VTE cohort (as the reference category). The demographic risk factors of VTE in the cancer inpatients were age between 36-50 years (OR: 1.16, 95% CI: 1.09-1.24), male sex (OR: 1.61, 95% CI: 1.54-1.69), and black (OR: 1.50, 95% CI: 1.41-1.60) and Hispanic (OR: 1.49, 95% CI: 1.40-1.59) ethnicities. Among the medical comorbidities, cancer inpatients with comorbid CHF had the highest risk of developing VTE (OR: 2.68, 95% CI: 2.30-3.12), followed by HIV/AIDS (OR: 1.67, 95% CI: 1.41-1.99), hypertension (OR: 1.23, 95% CI: 1.16-1.29), diabetes (OR: 1.16, 95% CI: 1.07-1.26), and chronic pulmonary disease (OR: 1.13, 95% CI: 1.05-1.22). In contrast, comorbidities like valvular disease, obesity, and drug abuse had a statistically non-significant association with VTE in cancer inpatients as shown in Table 2.

**Table 2:** Risk factors of venous thromboembolism in cancer inpatients HIV/AIDS: human immunodeficiency virus/acquired immunodeficiency syndrome

Variable	Odds ratio	95% confidence interval		P-value
		Lower limit	Upper limit	
Age				
18-35 years	Reference			
36-50 years	1.16	1.09	1.24	<0.001
Sex				
Male	1.61	1.54	1.69	<0.001
Female	Reference			
Race				
White	Reference			
Black	1.50	1.41	1.60	<0.001
Hispanic	1.49	1.40	1.59	<0.001
Other	1.22	1.12	1.32	<0.001
Comorbidities				
No comorbidities	Reference			
HIV/AIDS	1.67	1.41	1.99	<0.001
Congestive heart failure	2.68	2.30	3.12	<0.001
Valvular diseases	1.06	0.85	1.33	0.604
Chronic pulmonary diseases	1.13	1.05	1.22	0.001
Diabetes	1.16	1.07	1.26	<0.001
Hypertension	1.23	1.16	1.29	<0.001
Obesity	1.05	0.98	1.13	0.204
Drug abuse	1.05	0.91	1.20	0.508

The rate of in-hospital mortality was higher in the cancer inpatients with VTE (12%) as compared to the non-VTE group (2.1%). In the logistic regression model, a significantly higher odds of association between VTE and in-hospital mortality (OR: 3.87, 95% CI: 3.58-4.18) was observed as shown in Table 3.

**Table 3:** In-hospital mortality risk factor due to VTE in cancer inpatients VTE: venous thromboembolism

VTE	Rate, %	Odds ratio	95% confidence interval		P-value
			Lower limit	Upper limit	
No	2.1	Reference			
Yes	12.0	3.87	3.58	4.18	<0.001

## Discussion

We conducted a retrospective cohort study and found that 12% of adult cancer inpatients suffered from comorbid VTE. These results correlate with a Romanian study (n=6,592) conducted by Iorga et al., which found that 15% of the cancer patients had comorbid VTE [6]. We focused on the demographic factors (age, sex, and race) that may influence the risk of VTE in cancer. We found a 16% higher risk of VTE in middle-aged adults (35-50 years in age) compared to younger adults with cancer. This could be attributed to the fact that middle-aged adults are more likely to have medical comorbidities such as CHF, diabetes, hypertension, obesity, and chronic obstructive pulmonary disease (COPD), which lead to prolonged hospitalization, surgery, and immobility [9]. Women of childbearing age showed a higher risk due to pregnancy and oral contraceptive use, but after the age of 45 years, the risk is higher in men. This could explain the higher risk of VTE found in males in our study even though the underlying biologic cause remains unexplained [4]. We found that there is an increased risk of VTE in blacks and Hispanics compared to whites. This may be attributed to socioeconomic factors, access to healthcare, and racial disparities, such as blacks being less likely to receive direct oral anticoagulants [10]. Although blacks have a lower prevalence of factor V Leiden and other coagulation disorders compared to whites, they still maintain a higher risk. A previous study showed higher levels of hemostatic markers such as von Willebrand factor, factor VIII, and d-dimer in blacks. Also, factor VIII is higher, particularly in sickle disease, which is more commonly seen in blacks compared to whites [9].

There is a higher prevalence of medical comorbidities in cancer patients that leads to poor prognosis and lower survival rates, ultimately affecting their QOL and increasing healthcare costs. The common risk factors between comorbidities and cancer include cigarette smoking, alcohol use, lack of exercise, obesity, sedentary lifestyle, and poor dietary habits. Additional factors such as chronic infections, diabetes, and immunosuppressive conditions have also been known to predispose individuals to cancer [11].

Diabetes is a chronic condition known to release high circulating levels of insulin and insulin-like growth factors in the blood. This affects the programmed cell death called apoptosis, leading to the growth and proliferation of cells and increasing the probability of cancer. On the other hand, it is a prothrombotic condition that plays a major role in the development of hemostatic abnormalities, causing the endothelial injury of blood vessels through oxidative stress and activation of coagulation factors through gene transcription, promoting thrombosis through alteration in the fibrinolytic pathway [12,13]. This may explain the increased risk of VTE seen in our study in cancer patients with comorbid diabetes.

We also found a strong association between hypertension and VTE risk in cancer inpatients. It could be better explained in terms of several mechanisms to establish an association, such as damage to the venous valves due to endothelial injury, hypercoagulability, and activated platelets. Fibrinogenesis leading to venous stasis causes slow venous flow and the presence of superimposed coagulation mediated in hypertensive states places the patient at high risk of the inflammatory state of VTE [14]. Also, cancer patients with comorbid CHF had the highest risk of VTE (by 168%). This could be due to cardiovascular risk factors including smoking, obesity, hypertension, dyslipidemia, and diabetes. There exist several mechanisms related to the Virchow's triad contributing to the pathogenesis of thrombosis including altered blood flow to end organs due to poor cardiac output, poor contractility of chambers, and endothelial dysfunction in cells of the chamber leading to abnormalities in hemostasis and platelet activation [15]. There is a high incidence of venous thrombosis in chronic inflammatory lung disease due to endothelial injury-causing activation of platelets and coagulation factors, leading to thrombosis in veins [16]. In our study, we found that chronic pulmonary disease increases the risk of VTE by 13% in cancer patients.

HIV is a known prothrombotic condition with the affected individuals having a 2-10-fold increased risk of VTE when compared to the general population [17]. This is further supported by our study as we found HIV/AIDS increased the risk of VTE by 67% in cancer patients. Although the underlying pathophysiology is unknown, the increased risk may be related to a low cluster of differentiation 4 (CD4) cells (i.e., less than 200) leading to a hypercoagulable state along with deficiency of protein S, protein C, and antithrombin, and an increase in homocysteine levels. HIV-infected patients had a prevalence rate of protein S deficiency ranging from 27% to 76%, and of those, 12% had VTE [17].

We found a higher rate and risk of all-cause in-hospital mortality in cancer patients with VTE compared to those without VTE. Malignancy is associated with the compression of veins resulting in venous stasis, thereby promoting venous thrombosis. The development of VTE is initiated in the valve sinus, making the site prone to thrombosis due to the abnormal and reduced blood flow, reduced shear stress, and hypoxia leading to an intact but dysfunctional endothelium. In addition, platelets and leukocytes tend to become trapped in valve pockets. In cancer patients, tumors can compress veins, resulting in venous stasis, thereby promoting thrombosis and increasing mortality risk [5].

This study has some limitations. Since this was a retrospective, cross-sectional study, we could not find a causal relationship between the diagnosis of VTE and cancer patient mortality. This could be due to the lack of patient-level clinical information and the inpatient's diagnoses being based on diagnostic codes, which may have led to underreporting of comorbidities, preexisting conditions, and past diagnoses in medical records, such as prior VTE. Also, we could not include comorbidities like chronic infections and hereditary thrombophilia, which may impact the risk of VTE in cancer. The strength of our study is that we used NIS, a population-based dataset that is nationally representative, which included systemic and temporal factors. We utilized a large patient sample through a uniform collection of patient records, ensuring that the results were generalizable to the inpatient population. All of the information provided has a low risk of being distorted because it is coded and reported by different practitioners.

## Conclusions

In our study, VTE was predominantly seen in middle-aged males, and blacks and Hispanics suffering from cancer. The VTE risk was significantly higher in cancer patients with comorbid CHF (by 168%), HIV/AIDS (by 67%), hypertension (by 23%), diabetes (by 16%), and chronic pulmonary disease (by 13%). Also, the rate of all-cause in-hospital mortality was higher in cancer inpatients with VTE (12%), thereby increasing the risk of inpatient death by four times. Molecular mechanisms linked to the increased rate of tumor-associated thrombosis have been studied over the past few years, but significant gaps remain in our understanding of the causes and best approaches to thromboprophylaxis in cancer.
